# Biomarker expression patterns that correlate with high grade features in treatment naive, organ-confined prostate cancer

**DOI:** 10.1186/1755-8794-1-1

**Published:** 2008-01-31

**Authors:** Timothy J McDonnell, Nikhil S Chari, Jeong Hee Cho-Vega, Patricia Troncoso, Xuemei Wang, Carlos E Bueso-Ramos, Kevin Coombes, Shawn Brisbay, Remigio Lopez, George Prendergast, Christopher Logothetis, Kim-Anh Do

**Affiliations:** 1 Department of Hematopathology, The University of Texas MD Anderson Cancer Center, 1515 Holcombe Blvd, Houston, Texas 77030 USA.; 2 Department of Biostatistics, The University of Texas MD Anderson Cancer Center, 1515 Holcombe Blvd, Houston, Texas 77030 USA.; 3 Department of Pathology The University of Texas MD Anderson Cancer Center, 1515 Holcombe Blvd, Houston, Texas 77030 USA.; 4 Department of Genitourinary Medical Oncology The University of Texas MD Anderson Cancer Center, 1515 Holcombe Blvd, Houston, Texas 77030, USA.; 5 Lankenau Institute for Medical Research, Wynnewood, PA 19096, USA.

## Abstract

**Background:**

The early detection of prostate cancer has resulted in an increase in the number of patients with localized prostate cancer and has paralleled the reported reduction in prostate cancer mortality. The increased rate of detection of patients with localized prostate cancer may also increase the risk of potentially morbid therapy in a patient with indolent cancer. Defining the biomarker correlates of prostate cancer virulence will facilitate the appropriate application and development of therapy for patients with early disease.

**Methods:**

A 255 core prostate cancer tissue microarray (TMA) from 47 prostatectomy specimens with organ confined tumor was constructed. Prostate cancer foci of transition and peripheral zone origin were represented on the TMA. Further, replicate cores of the two Gleason grades comprising the Gleason score, representative of Gleason scores 5-9, were arrayed from each prostatectomy specimen. Standard immunohistochemical techniques were used to assess expression of nine, cell death and cell cycle regulatory proteins implicated in the pathogenesis of prostate cancer (bax, bcl-2, bcl-x_L_, bin1, CD95, mdm2, p21, p53, and NFkB).

**Results:**

The Spearman correlation coefficient revealed a strong correlation of bax, bin1, FAS, p65 and p21 expression with Gleason grade. Spearman correlation coefficients showed that expression of, bax and bin1, bax and MDM2, Bax and p21, and bax and p65 NFkB was highly associated. Other significant associations were identified between bin1 and p21, bin1 and MDM2, bin1 and p65 NFkB and between p21 and p65 NFκB. A model for predicting the biological potential of Gleason score 7 prostate cancer using multivariable logistic regression methods was developed. The findings also indicate that the profile of specific markers for Gleason grade 3 prostate cancer correlates with the overall context of the Gleason score.

**Conclusion:**

These data support the view that important molecular differences exist among and between the Gleason scores. Furthermore, there is significant molecular heterogeneity among prostatectomy specimens containing Gleason grade 3 cancer. This observation may have broader implications regarding the determination of risk among patients with prostate cancer that is currently considered to be of either good prognosis or unclear prognosis, i.e. Gleason score 7 tumors.

## Background

The prediction of outcome in prostate cancer would immediately benefit a portion of patients diagnosed with early stage prostate cancer detected because of the widespread application of PSA screening. Patients with aggressive disease would be encouraged to consider early intervention whereas those with cancer that are not likely to progress could safely be managed by watchful waiting. Current strategies apply morphologic criteria to approach this problem. The understanding of the molecular basis of prostate cancer provides an opportunity to develop an objective method to address these issues as well as the potential to provide insights into the mechanistic basis of prostate cancer progression. The dilemma of most patients with prostate cancer is that they are increasingly comprised of low volume Gleason score 7 tumors detected by routine screening thus exposing some patients to the risk of unnecessary intervention. In addition, this heterogeneity has made it difficult to perform efficient clinical investigation in these patients.

The development of tissue microarrays (TMA) represents a significant technical advance and enables a much higher throughput in the assessment of the clinical and prognostic significance of candidate biomarkers [1]. TMAs have the additional advantage of maximizing the utilization of frequently limited tissue samples. TMA technology is also expanding our conceptual approaches to the analysis of routinely acquired human tissue samples. TMAs can be designed to address specific clinical or pathologic questions of interest including tumor type, tumor progression, and response to therapy. TMAs are frequently incorporated into global genomic expression profiling studies of human tumors as a means to rapidly validate newly identified, and potentially informative, biomarkers [2-4].

Structured information obtained from tissue microarray analysis is analogous to that obtained from high density cDNA microarrays, "chips", now routinely used in expression profiling studies. Thus, as with cDNA arrays, it is possible to use the large amounts of data (routinely thousands of data points from multiple biomarkers) to develop statistical models appropriate to questions of clinical and biologic interest. With these issues in mind a 255 core TMA was created, representative of tumor foci from 47 prostatectomy specimens from patients with organ confined prostate cancer that received no other form of therapy. The array was constructed to address the speculation that variation in biomarker expression could define signatures associated with the zonal origin of tumors. It was further speculated that biomarker signatures could identify subsets of cancers within Gleason grades as defined by histopathology and provide a basis for more accurate prognostication. The microarray included replicate samples of the two Gleason grades comprising the Gleason score for each tumor focus arrayed. A selected number of cell death and cell cycle regulatory proteins were assessed using routine immunohistochemistry. Using this data, a model for predicting the biologic potential of Gleason score 7 prostate cancer using multivariable logistic regression methods was developed. These findings also indicate that the profile of specific markers for Gleason grade 3, a critical component of Gleason score 7, prostate cancer correlates with the overall context of the Gleason score. This suggests that the molecular profile of the Gleason grade 3 component varies, and correlates, with the overall Gleason score of the tumor focus and challenges the concept that all Gleason grade 3 prostate cancer components are clinically and biologically equivalent.

## Methods

### Tissue Acquisition and Construction of Tissue Microarray

The Beecher Instrument (Silver Spring, MD) tissue microarray apparatus was used to generate the TMA. An H&E stained section from each donor block was examined and the areas of interest identified and marked. A 10 mm thick paraffin block was prepared to be used as the recipient block. The corresponding H&E stained slide, or image transparency, was placed over the donor block and the area of interest aligned with the stylet. The sample information (i.e. address and source of the core within the microarray) was entered onto an array worksheet. Individual areas of the two Gleason grades comprising the Gleason score of the tumor focus were each sampled 3 times. The completed array represented a total of 50 donor blocks from 47 prostatectomy specimens and was comprised of 255 individual cores, each 0.6 mm in diameter. Each of the 50 donor blocks was representative of a tumor focus so that one tumor focus was arrayed for 44 of the prostatectomies and 2 tumor foci were arrayed for 3 of the prostatectomies. All patient samples were collected in accordance with established practices and procedures and were approved by the Internal Review Board at UT- MD Anderson Cancer Center, Houston, TX.

### Immunohistochemical Techniques

All immunohistochemical sections were stained with a Dako Autostainer utilizing standard immunohistochemical strepavidin-biotin techniques. Sections were first deparafinized and then rehydrated. Antigens were retrieved using the Dako Target Retrieval solution in conjunction with steam. Sections were then treated to block endogenous peroxidase, avidin, biotin and protein. After blocking, sections were incubated with specific primary antibody at the appropriate concentration for 60 minutes at room temperature. Primary antibodies used in this study were bax, bcl-x_L_, bcl-2, bin-1, CD95 (Fas/APO-1), MDM2, p21, p65 (NFkB), and p53 (DAKO, Calbiochem, Santa Cruz, Alexis, R & D). The primary antibody 2F11 is a 'pan-Bin1' antibody that recognizes all the Bin1 splice isoforms that have been described [5]. Sections were rinsed and then incubated with a biotinylated universal secondary antibody followed by horseradish peroxidase labeled strepavidin both at room temperature for 15 minutes each (Dako's LSAB+ kit). The 3,3 diaminobenzidine substrate was then applied for 15 minutes at room temperature. Sections were counterstained with Mayer's hematoxylin, dehydrated, cleared and coverslipped. The appropriate positive and negative controls were used for each antibody.

### Tissue Array Database (TAD) and Image Analysis

The BLISS Image Analysis Workstation was used to scan H&E and immunohistochemically-stained TMA slides. Using the Webslide server these slides were scored and entered directly into the TAD [6,7]. Also, an Active X component in the TAD was developed that allows the image scanned on the BLISS system to be linked directly with the core in the TAD. A LaCie RAID system, approximately 900 GB of redundant storage capacity, was used to store all of the BLISS image files.

### Statistical Analysis

Spearman correlation coefficients were used to assess the association between different biomarkers' expression, or the association between biomarker expression and Gleason score. Due to potential association among the multiple cores taken from the same patient tumor sample, a linear mixed effect model was fit to assess the association between each biomarker's expression and zone of tumor origin. Bonferroni correction was applied to account for multiple testings. A GEE (Generalized Estimation Equations) model was fit to simultaneously assess the predictive effects of biomarker expressions on the probability of having poor prognosis among patients having Gleason score > 7 or < 7. Stepwise model selection was carried out and the final fitted model was used to compute the predictive probabilities for Gleason score 7 tumors to have poor prognosis.

The biomarker expression levels were categorized as 0, 1, 2, 3 and 4. A composite score was calculated for each Gleason score/Gleason grade combination group based on the number of cores having each biomarker expression level. The composite scores for each Gleason score/Gleason grade combination were then plotted against the 9 biomarkers in a "heat" map, where bright red corresponding to the highest expression level and bright green corresponding to the lowest expression level.

## Results

### Construction of Prostate Tissue Microarray

A 255 core tissue microarray (TMA) was constructed from formalin-fixed and paraffin-embedded prostatectomy specimens obtained from patients with prostate cancer as described in the methods. The TMA was representative of the spectrum of prostate cancer Gleason grades, ranging from Gleason Score 5 to Gleason Score 9. A total of 50 tumor foci (one focus from 44 specimens and 2 foci from 3 specimens) including 35 of peripheral zone and 15 of transition zone origin were included in the array. A summary of the tissue core composition of the TMA is presented in Tables 1 and 2.

**Table 1:** Gleason Score Distribution of TMA

Gleason Score	Gleason Grade	Anatomic Zone	Blocks (n)
5	2 + 3	TZ	5
	3 + 2	TZ	5
6	3 + 3	TZ	5
		PZ	10
7	3 + 4	PZ	5
	4 + 3	PZ	5
8	4 + 4	PZ	9
	3 + 5	PZ	1
	5 + 3	PZ	1
9	4 + 5	PZ	4

**Table 2:** Distribution of Cores by Gleason Grade on TMA

**Core Gleason Grade**	**Core Source (Gleason Score)**	**n**
2	**2 **+ 3	15
	3 + **2**	15
3	2 + **3**	15
	**3 **+ 2	15
	**3 **+ **3**	45
	**3 **+ 4	15
	4 + **3**	15
	**3 **+ 5	3
	5 + **3**	3
4	3 + **4**	15
	**4 **+ 3	15
	**4 **+ **4**	54
	**4 **+ 5	12
5	3 + **5**	3
	**5 **+ 3	3
	4 + **5**	12

		**Total Cores (n) 255**

### Structuring of Immunohistochemical Data

The expression of selected biomarkers associated with cell cycle progression and/or cell death sensitivity was assessed using routine immunohistochemical methodologies. These markers included bax, bcl-2, bcl-x_L_, bin-1, CD95 (Fas/APO-1), MDM2, p21^waf1/cip1^, p53, and p65 (NFkB). In order to facilitate the statistical analysis of the immunohistochemical information of the various markers the data was structured in the following manner. The percentage of tumor exhibiting detectable levels (i.e., involvement) of a specific marker was scored 0 (no detectable staining above background), 1 (1-25% of the tumor cells exhibiting detectable staining), 2 (25-75% of the tumor cells exhibiting detectable staining), or 3 (> 75% of the tumor cells exhibiting detectable staining). The intensity of staining for the individual markers, if detectable, was scored as either low (1) or high (2). Staining intensity was assessed relative to internal positive controls, such as basal epithelial cells or lymphocytes present on the array, or tissue controls used to establish the appropriate antibody titrations. Data analysis revealed that there was no significant difference attributable to subcellular distribution of the biomarkers assessed in this study. In order to incorporate both the involvement and the intensity information for each biomarker a new variable, "expression" was generated, which was defined as the following:

Expression = 0 if involvement = 0 (intensity = 0 by definition)

= 1 if involvement = 1

= 2 if involvement = 2

= 3 if involvement = 3 and intensity = low

= 4 if involvement = 3 and intensity = high

After careful observation of the patterns shown in our original data, a new variable, expression, was defined (Table 3). The data set had a special feature in that the intensity information only became relevant in defining ''expression'' when involvement = 3. When involvement = 0, 1 or 2, all cores had ''low'' intensity, except 3 cores when involvement = 2.

**Table 3:** The number of cores by involvement and intensity category.

		Intensity	
		
		Low	High
Involvement	0	1202	0
	1	70	0
	2	159	3
	3	547	254

### Statistical Analysis and Data Modeling of Biomarkers

The prostate cancer TMA was constructed to address several issues of both biologic and clinical interest. It was first of interest to determine the frequency of individual biomarker expression and to determine the extent to which individual biomarkers correlated with the prostate zone of tumor origin, i.e. peripheral versus transition zone within Gleason grade 3+3 tumors (Table 4). Bcl-2 and p53 exhibited no detectable levels of expression in this subset of Gleason grade 3+ 3 tumors and were therefore not included in Table 4. For each of the other biomarkers, a linear mixed effect model was fit for the biomarker expression, using the zone of origin as a fixed effect and patient as a random effect. The mixed model also took into account the potential association among multiple cores within the same subject through modeling the variance-covariance matrix of the residuals. The model suggested that there was no significant difference in CD95 or bcl-x_L _expression between peripheral zone and transition zone tumors (p = 0.61 and 0.12, respectively). Among bax (p < 0.0001), bin-1 (p = 0.005), p21 (p = 0.0008), mdm2 (p = 0.02) and p65 NFkB (p = 0.02), the model suggested that the expressions level in transition zone was significantly lower than that of the peripheral zone. The Bonferroni method was used to adjust for multiple comparisons (i.e., using a cutoff value of 0.05/7 = 0.007), only bax and p21 remained to show significant differences between the peripheral and transition zone with regards to biomarker expression.

**Table 4:** The fitted linear mixed effect models for each biomarker expression within Gleason grade 3+3 tumors, where zone of origin (TZ vs. PZ) was fitted as a fixed effect and patient as a random effect.

**Marker**	**Variable**	**Coefficient**	**SE**	**P-value**
Bax	Intercept	2.73	0.18	-
	TZ (vs. PZ)	-2.27	0.32	**< 0.0001**
				
Bcl-XL	Intercept	3.58	0.15	-
	TZ (vs. PZ)	-0.25	0.15	0.12
				
Bin1	Intercept	2.13	0.25	-
	TZ (vs. PZ)	-1.47	0.44	**0.005**
				
FAS	Intercept	0.27	0.22	-
	TZ (vs. PZ)	-0.20	0.39	0.61
				
MDM2	Intercept	2.81	0.60	-
	TZ (vs. PZ)	-2.81	1.04	**0.02**
				
P21	Intercept	2.67	0.28	-
	TZ (vs. PZ)	-2.13	0.49	**0.0008**
				
P65	Intercept	1.67	0.37	-
	TZ (vs. PZ)	-1.67	0.63	**0.02**

**Figure 1:**
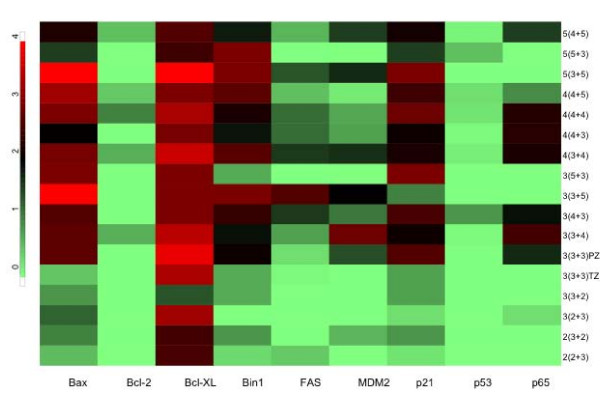
Plot of biomarker expression vs. Gleason grade. Bright red corresponds to the highest expression level, black corresponds to moderate expression and light green is the lowest expression.

The Spearman's correlation coefficient was used to assess potential significant associations between Gleason grades and biomarker expression for each of the nine biomarkers assessed. The array was comprised of 30 cores of grade 2 tumor, 111 cores of grade 3 tumor, 96 cores of grade 4 tumor, and 18 cores of grade 5 tumor. Using the median biomarker expression within each subject, there was no significant association between the level of mdm2, bcl-x_L _or p53 expression and Gleason grade (p = 0.19, p = 0.27 and p = 0.75, respectively). However, all of the six remaining biomarkers exhibited a significant association with Gleason grade. This association was strongest for bax (r = 0.62, p < 0.0001) and p21 (r = 0.57, p = 0.0002) with each marker exhibiting increasing expression with corresponding increases in Gleason grade. The majority of the cores for Fas, and p65 exhibited no detectable expression, however, when expressed these markers were associated with higher Gleason grade (p = 0.01 and p = 0.002, respectively). The "heat map" enabled a visual representation of the variation in individual biomarker expression associated with Gleason grade (Figure 1).

The Spearman correlation coefficient was computed to identify significant associations between pairs of biomarkers (Table 5). The median biomarker expression level within each subject was used for this calculation. This analysis demonstrated that the expression of bax and bin1, bax and MDM2, bax and p21, and bax and p65 NFkB was highly associated. Other significant associations were identified between bin1 and p21, bin1 and MDM2, bin1 and p65 NFkB and between p21 and p65 NFkB. Similar associations were identified when using the mean, instead of the median biomarker expression level within each subject to calculate the Spearman correlation coefficient.

**Table 5:** Spearman correlation among biomarkers with regards to the median expression levels within subject.

	**Bax**	**Bcl-2**	**Bcl-XL**	**Bin1**	**FAS**	**MDM2**	**p21**	**p53**	**p65**
**Bax**	1	0.42	0.47	**0.78**	0.39	**0.57**	**0.68**	0.33	**0.58**
**Bcl-2**		1	0.14	0.01	0.25	0.08	0.44	0.21	0.31
**Bcl-XL**			1	0.48	0.07	0.25	0.39	-0.08	0.32
**Bin1**				1	0.37	**0.54**	**0.63**	0.27	**0.62**
**FAS**					1	0.20	0.19	0.38	0.37
**MDM2**						1	0.38	0.38	0.49
**p21**							1	0.19	**0.63**
**p53**								1	0.34
**p65**									1

Univariate and multivariable logistic regression models were used to predict the biologic potential of Gleason score 7 (either 3 + 4 or 4 + 3) tumors using a profile of the biomarkers included in this study. For the purposes of this study Gleason score 5 and 6 tumors were considered clinically indolent (good prognosis) and Gleason score 8 and 9 tumors were considered clinically aggressive (poor prognosis). Our goal was to assess whether Gleason score 7 tumors are more similar to Gleason score < 7 (good prognosis) tumors or closer to Gleason score > 7 (poor prognosis) tumors, based on a fitted model using biomarker expression profiles. First, a generalized estimation equation (GEE) model was fit for the patients with Gleason score not equal to 7. The response variable was equal to 1 if the patient had poor prognosis (i.e., Gleason score > 7), and 0 otherwise. The GEE method takes into account the association among the multiple cores obtained from the same patient through modeling a working correlation matrix. In our case, the working correlation matrix was specified to be "unstructured" (UN). After a stepwise model selection procedure, a final fitted model was determined (Table 6). Based on this model, Bax involvement = 3, FAS involvement > 0, p21 involvement = 3 was associated with an increased odds of having poor prognosis, and Bcl-XL intensity = high was associated with a reduced odds of having poor prognosis.

**Table 6:** Fitted GEE model for predicting poor prognosis (GS > 7) in patients with Gleason score < 7 or > 7.

**Effect**	**Coefficient**	**Standard Error**	**95% Confidence Limits**		**P-value**
Intercept	0.32	0.07	-	-	-
Bax involvement = 3	0.04	0.02	0.002	0.08	0.04
FAS involvement = 1, 2 or 3	0.05	0.02	0.02	0.08	0.001
p21 involvement = 3	0.05	0.01	0.03	0.07	< .0001
Bcl-xl intensity = high	-0.05	0.02	-0.08	-0.01	0.01

The model shown in Table 6 was used to predict the probability of having poor prognosis for the patients with Gleason score 7 tumors (n = 10 patients). The results indicate that the predicted probability of poor prognosis in those Gleason score 7 patients (i.e., the within-patient mean predicted probability) ranges from 0.340 to 0.467. In contrast, in patients with Gleason score greater than 7, the predicted probability for poor prognosis ranges from 0.386 to 0.417, while in patients with Gleason score less than 7, the predicted probability ranges from 0.312 to 0.376, except for one patient whose predictive probability is 0.426. If 0.386 is used as the cutoff value for classifying poor prognosis, then 6 out of the 10 patients with Gleason score 7 would be classified as having poor prognosis.

Subsequently, it was of interest to determine whether all Gleason grade 3 tumor areas were equivalent with respect to the biomarker expression profile, or whether subsets could be defined which exhibited differences in biomarker expression. The Gleason scores containing '3' include 2+3, 3+2, 3+3, 3+4, 4+3, 3+5 and 5+3. The association between Gleason score and biomarker expression was assessed using the Spearman correlation coefficient. Again, the median biomarker expression was used in each patient. This analysis revealed significant differences between Gleason grade 3 biomarker signatures that varied within the context of the Gleason score (Table 7). This finding implies that, with respect to the expression of the biomarkers assessed in this study, not all Gleason grade 3 tumor areas are equivalent. Further, the results indicate that the Gleason grade 3 signatures obtained correlated with the corresponding clinically indolent or clinically aggressive categories of the overall Gleason score.

**Table 7:** The association between the median biomarker expression within subject and the Gleason scores in patients having Gleason Grade 3.

**Biomarker**	**Spearman correlation coefficient**	**P-value**
Bax:	0.58	0.0007
Bcl-XL	0.15	0.38
Bcl-2	0.15	0.39
Bin1	0.45	0.009
FAS	0.44	0.01
MDM2	0.30	0.08
P21	0.60	0.0005
P53	0.23	0.18
P65	0.44	0.01

## Discussion

The use of TMAs as a strategy to validate and characterize candidate biomarkers is gaining widespread acceptance as an alternative to more traditional immunohistochemical methods. Less widely appreciated, is that TMAs also facilitate a rapid assessment of biomarker expression in larger cohorts of tissue and are thereby amenable to the development of statistical models based on structured immunohistochemistry information [8]. In this regard it is noteworthy that while there is general acknowledgement of the prognostic value of the Gleason score there is also agreement on the limitations of morphology in the prediction of clinical outcome for individual patients. In part this limitation may be attributable to the considerable interobserver variability in the assignment of Gleason grade [9].

In this study, a 255 core TMA comprising the spectrum of low grade to high grade Gleason scores of organ confined tumors was used to address several questions of clinical and pathological interest based on statistical modeling of the expression of selected biomarkers. These biomarkers were comprised of well characterized cell cycle (p53, p21, mdm2) and cell death (p53, bax, bcl-x_L_, bcl-2, bin1, CD95, NFkB) regulators variably expressed by human prostate cancers [5,10-16]. Available evidence suggests that at least several of these proteins may be of predictive value [15,17-21]. While the scope of this study is focused on a small number of selected biomarkers, the analysis is based on nearly 7,000 data elements and highlights the critical issues of data management and distribution. A tissue array database (TAD) was, therefore, developed to facilitate the structuring, storage, and distribution of biomarker information. TAD consists of an Active Server Page web interfaced with a relational database that automates recording biomarker scores and links the structured data with clinical and pathologic information. TAD is an open source application that can be installed locally.

It is widely appreciated that Gleason score 7 prostate cancers in aggregate have an intermediate prognosis but are clinically heterogeneous despite their morphologic similarities and, conversely, a significant number of Gleason score 6 prostate cancers, despite their overall good prognosis, exhibit clinically aggressive behavior [22]. Clearly, a biomarker signature capable of contributing to the predictive value of accepted histopathologic information would be of considerable value. As a first step to realizing this goal we modeled expression of selected proteins on a tissue microarray comprising the spectrum of Gleason score organ confined tumors. Our analysis suggests that four markers (bcl-x_L_, Fas, bax, and p21) provided potentially important information predictive of clinically aggressive behavior. The findings also indicate, that tumors of equivalent Gleason grade although morphologically similar, have varying patterns of biomarker expression that correlate with the Gleason score of the tumor.

Represented in the TMA were 15 cases of Gleason score 6 (3 + 3) carcinoma consisting of tumors of transition zone (5 cases) or peripheral zone (10 cases) origin defined by established histopathological criteria, that enabled an assessment of the potential variability of individual biomarker expression as a function of zonal origin of the tumor. Tumors of peripheral zone origin that exhibited secondary involvement were excluded. It is noteworthy that bax, bin1, mdm2, p21, and p65 NFkB were all expressed at significantly higher levels in peripheral zone tumors compared to transition zone tumors of equivalent Gleason score. Although establishing the biological, or potential clinical, significance of these observations is beyond the scope of the current study it is of interest that transition zone prostate carcinomas are both less common and typically indolent compared to their counterparts arising from the peripheral zone [23,24]. Additionally, it has been speculated that transition zone and peripheral zone carcinoma may arise from different precursor lesions [23]. Although it has not been previously established, it is, therefore, not completely unanticipated that carcinomas arising from the peripheral zone would exhibit patterns of biomarker expression that differ from those arising in the transition zone.

In pairwise comparisons of the expression of the nine biomarkers using Spearman correlation coefficients several combinations of biomarkers exhibited highly concordant expression. There were significant associations between bcl-x_L _and bax, bax and bin1, and bax and p21. Although proapoptotic and antiapoptotic proteins of the bcl-2 gene family have been shown to be coordinately regulated [25], as well as bax and p21 [26], an association between bax and bin1 has not been described. It would be of interest to determine the extent to which apoptosis, or tumor suppressor activity, mediated by bin1 may be dependent on bax.

The results of this study suggest opportunities for future laboratory-based mechanistic studies. Finally, biostatistical strategies are presented that suggest it may be possible to use a panel of biomarkers to provide predictive information about Gleason score 7 prostate cancers. Ultimately, these findings will need to be expanded to determine, prospectively, whether they will prove of clinical utility beyond current histopathologic variables including Gleason grade and tumor stage. It is anticipated that multiparametric analyses can be performed on limited tumor specimens and will lead to improved clinical investigation and better validation of mechanistic concepts. Incremental advances in prostate cancer therapy will likely rely on integration of validated molecular information with the widely applied morphologic characterization of prostate cancer. Integration of this information with other prognostic variables including tumor stage and pretreatment PSA levels will be necessary to establish its ultimate utility. This will necessitate rigorous validation in clinically annotated specimens with long-term follow up.

## Conclusion

In general improved models of prediction of disease outcome are required to allow the clinician to design more appropriate treatment modalities for prostate cancer patients. This study presents data supporting the theory that significant differences in molecular signatures exist among a large group of Gleason grade 3 cancers. Furthermore, a biostatistical model was developed using a limited number of molecular markers that may enable more accurate prediction of risk of prostate cancer progression. These findings support a more detailed study of a larger cohort of clinically annotated tissue from prostate cancer patients.

## Abbreviations

TMA, Tissue MicroArray; ROC, Receiver Operating Characteristics; TAD, Tissue array database; TZ, Transition Zone; PZ, Peripheral Zone.

## Competing interests

The author(s) declare that they have no competing interests.

## Authors' contributions

TJM conceived the study, participated in its design and co-ordination, was involved in data acquisition and manuscript preparation. NSC was involved in co-ordination and manuscript preparation. CBR and JHC were involved in data acquisition. XW, KD, and KC were involved in data analysis and statistical modeling. SB and RL were involved in immunohistochemical staining and imaging. PT provided annotated tissue and aided in data acquisition. GP was involved in reagent generation and manuscript preparation. CL was involved in manuscript preparation. All the authors have read and approved the final manuscript.

## Pre-publication history

The pre-publication history for this paper can be accessed here:


